# The Relationship Between Affective and Obsessive-Compulsive Symptoms in Internet Use Disorder

**DOI:** 10.3389/fpsyg.2021.700518

**Published:** 2021-08-12

**Authors:** Tania Moretta, Giulia Buodo

**Affiliations:** Department of General Psychology, University of Padova, Padova, Italy

**Keywords:** Internet use disorder, Internet addiction, obsessive-compulsive symptoms, depression, anxiety, diagnostic criteria, behavioral addiction, hoarding

## Abstract

We investigated the relationships and diagnostic power of symptoms associated with affective disorders, obsessive-compulsive disorder, and drug addictions on Internet use disorder. Moreover, we tested whether Internet use disorder is characterized by a specific network of symptoms. One-hundred-and-four young adults (78 women) were assessed in laboratory using self-report measures of Internet addiction, alcohol use disorder, cannabis abuse, depression, anxiety, and stress symptoms, impulsiveness, and obsessive-compulsive symptoms. Only hoarding, obsessing, and depression symptoms were positively linked to Internet use disorder severity, with hoarding having greater power and accuracy than other obsessive-compulsive and affective symptoms. Only individuals with mild-moderate Internet use disorder were characterized by a network of strong and positive associations of affective and obsessive-compulsive symptoms. These findings may encourage future longitudinal studies aimed at identifying potential clinical criteria for the diagnosis of Internet use disorder and treatment targets.

## Introduction

Despite the growing number of studies on Internet use disorder (IUD), there is not yet an agreement on the conceptualization of Internet-related problematic behaviors. Of note, only Internet gaming disorder (IGD), one of the sub-types of IUD, has been recently included in the research appendix of the fifth edition of the DSM (DSM-5, American Psychiatric Association, 2013), however, the DSM-5 itself noted that more studies are needed, both in the context of IGD as well as more general IUD, to confirm and/or update the proposed criteria.

Given these issues and the current absence of IUD in any official classification of mental disorders, in the present work we endorse one of the most recent definitions of IUD, i.e., a condition involving excessive or poorly controlled urges and behaviors relating to Internet use that lead to subjective distress and/or interference in major areas of life functioning. It is a heterogeneous construct that may include a multitude of features relating to sexual, social networking, and gaming behaviors (Banz et al., 2016). Considering the negative consequences of IUD on affected individuals' life and their relevance for public health, identifying the diagnostic criteria for IUD should be one of the main aims of research in this field to improve reliability across studies and to develop effective treatment approaches and prevention measures (Kuss and Lopez-Fernandez, 2016).

To identify and conceptualize diagnostic criteria for IUD, several studies assessed specific psychological characteristics of individuals with IUD (e.g., Peterka-Bonetta et al., 2019). Among these studies, it has been mainly reported that individuals with IUD are more likely to have symptoms related to the *affective* and *impulsive-compulsive domains*, and *substance addictions* (e.g., Anderson et al., 2017; Bernal-Ruiz et al., 2017). The interaction between low mood regulation and high stress reactivity (*affective domain*) and high impulsivity/poor inhibitory control (*impulsive-compulsive domain*) has been suggested to be a key aspect of both behavioral addiction and IUD. Specifically, studies that investigated *affective domain*-related alterations in IUD found that individuals who are highly responsive to stressors and employ impulsive coping strategies would be more inclined to use the Internet for mood regulation. Indeed, they seem to hold false beliefs about the power of the Internet to regulate their negative mood (Brand et al., 2019). Similarly, studies that investigated the *impulsive-compulsive domain*-related alterations in IUD found that impulsivity levels are positively associated with- or are predictive of IUD (e.g., Shokri et al., 2017), suggesting that impulsivity may be a core personality trait in problematic Internet users. An association between IUD and obsessive-compulsive symptoms has been also observed (e.g., Stavropoulos et al., 2016). Interestingly, it has been argued that individuals with IUD would be characterized by alterations of reward-related processing and behaviors that would lead to the co-occurrence of symptoms associated with obsessive-compulsive disorder (OCD) and substance addictions (Brand et al., 2019). Studies that investigated the relationship between *substance addiction* and IUD found that adolescents with IUD are more likely to use substances and, among substance abuse, problematic alcohol use has been suggested to share similar characteristics and predictors with IUD (Gámez-Guadix et al., 2015). Similarly, a link between IUD and cannabis use has been found, with most studies reporting small to medium positive associations between IUD and cannabis use (Lanthier-Labonté et al., 2020).

To the best of our knowledge, only a few studies to date have investigated together the potential links between IUD and alterations related to the *affective and impulsive-compulsive domains*. Specifically, it has been suggested that young people with obsessive-compulsive symptoms could become excessive Internet users to modulate affective symptoms (Bernal-Ruiz et al., 2017). However, associations between IUD, alterations related to the *affective domain*, that may drive problematic Internet use behaviors, and the *impulsive-compulsive domain*, that may hinder inhibition of that behaviors should be further explored, together with *substance abuse*, that may co-occur with, and be a risk factor for, IUD. To address this gap, a comprehensive model involving alterations in the *affective domain* (depression, anxiety, stress symptoms), in the *impulsive-compulsive domain* (impulsivity and OCD-related symptoms), and the *abuse of substances* (alcohol and cannabis abuse) is presented and tested as a preliminary study that may encourage future longitudinal investigations. Moreover, to our knowledge, this is the first study to explore the existence of a specific network of relationships between alterations related to *affective* and *impulsive-compulsive domains* and *substance abuse* in individuals with IUD.

The goals of this preliminary study were: (i) to investigate in a comprehensive model the link between IUD and depression, anxiety, and stress (*affective domain* variables), impulsivity and obsessive-compulsive symptoms (*impulsive-compulsive* domain variables), and alcohol and cannabis abuse (*substance abuse* variables); (ii) to explore the efficiency of *affective domain, impulsive-compulsive domain*, and *substance abuse* variables in discerning individuals with IUD-related problems from controls; (iii) to examine whether a specific network of associations among these variables characterizes individuals with IUD-related problems vs controls. The following hypotheses were formulated: (i) *affective domain, impulsive-compulsive domain*, and *substance abuse* variables would each be positively linked to IUD severity; (ii) because the efficiency of study variables to discern groups was an empirical question to be answered by this study, we did not make specific predictions regarding how well each variable would discern individuals with IUD-related problems from controls; (iii) individuals with IUD-related problems would be characterized by positive stronger intercorrelations between *affective domain, impulsive-compulsive domain*, and *substance abuse* variables than controls.

## Methods

### Participants

**Figure 1:**
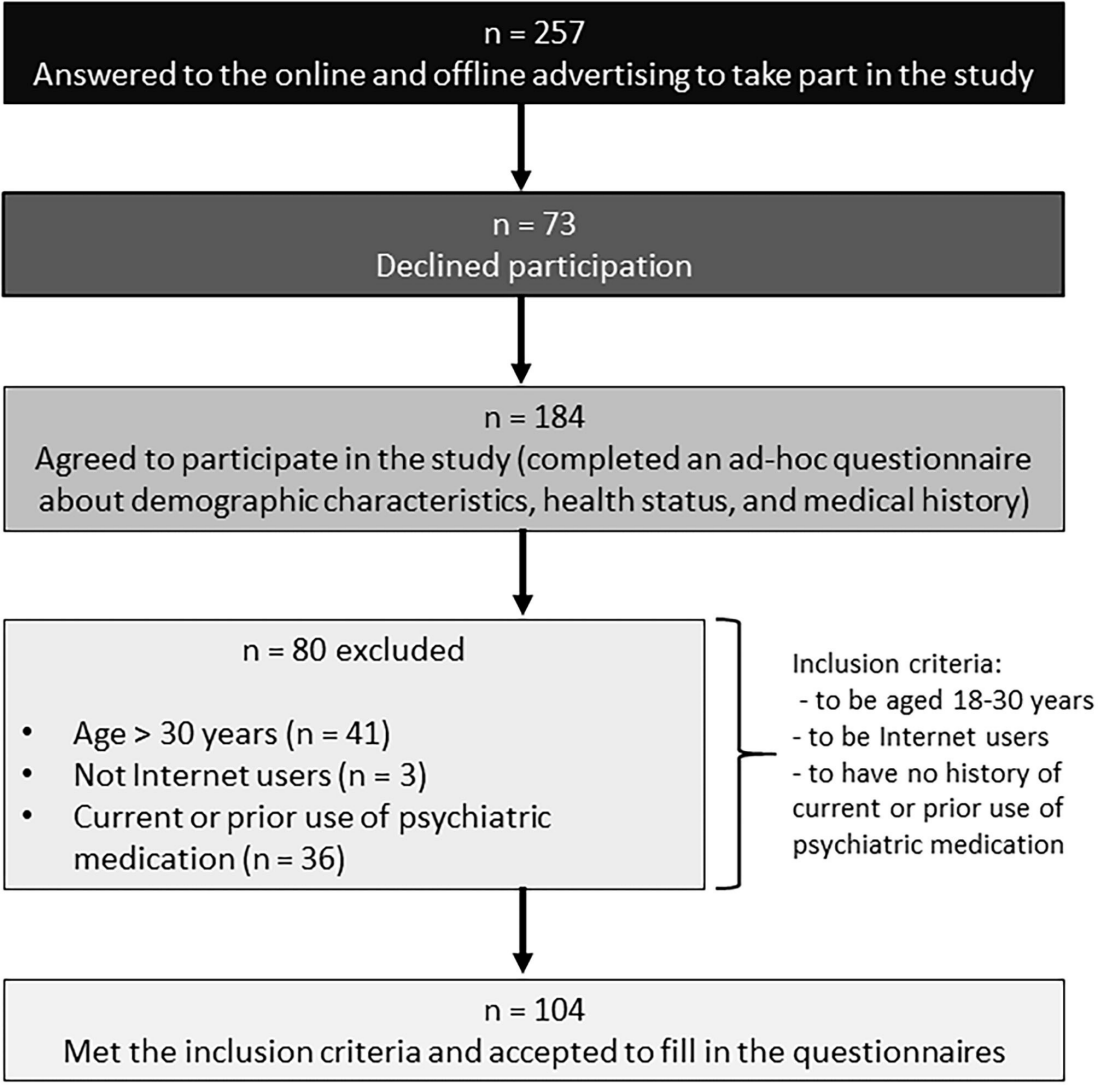
Recruitment flow chart.

Participants were recruited via online and offline advertising. To be eligible for inclusion in the study, participants were required to be aged 18–30 years, to be Internet users, and to have no history of current or prior use of psychiatric medication (e.g., antipsychotics, antidepressants, mood stabilizers). Those who met the inclusion criteria and accepted to participate in the study were administered a paper-and-pencil version of the Italian versions of the Internet Addiction Test (Ferraro et al., 2007), the Depression Anxiety-Stress Scales (Bottesi et al., 2015), the Barratt Impulsiveness Scale (Fossati et al., 2001), the Obsessive-Compulsive Inventory-Revised (Sica et al., 2009), the Alcohol Use Disorders Identification Test (Addolorato et al., 1999), and the Cannabis Abuse Screening Test (Bastiani et al., 2013). More details about the recruitment process are reported in Figure 1.

Given that the present study is the first to describe in a single model the relationships of *affective domain, impulsive-compulsive domain*, and *substance abuse* variables with IUD severity, there was no related effect size to choose from for formal power analysis. The present study has been conducted as a first hypothesis testing and should be used to design larger confirmatory studies.

A total sample of 104 young adults (*F* = 78, mean age = 23.21 ± 2.97, mean years of education = 16.76 ± 2.11) participated in this study and, based on their Internet Addiction Test scores, 70 participants were classified as control group (*F* = 54, mean Internet Addiction Test score = 34.69 ± 7.30, mean age = 23.13 ± 2.89, mean years of education = 16.79 ± 2.21, sleep hours = 7.27 ± 0.71, daily cigarettes consumption = 1.73 ± 3.49), and 34 as individuals with mild to moderate IUD (*F* = 24, mean Internet Addiction Test score = 57.68 ± 7.00, mean age = 23.38 ± 3.17, mean years of education = 16.71 ± 1.90, sleep hours = 7.15 ± 0.80, daily cigarettes consumption = 1.97 ± 4.03). Internet Addiction Test scores among individuals with mild-moderate IUD were significantly higher than among those in the control group (*t* = 15.3, *p* < 0.001). No differences between groups were found for age, sex, years of education, sleep hours, and cigarette consumption.

The present study was carried out with the adequate understanding and written consent of the participants in accordance with the Declaration of Helsinki. The study was approved by the local Ethics Committee.

### Self-Report Measures

The Internet Addiction Test (IAT; Young, 1998; Italian version by Ferraro et al., 2007) was used to assess IUD severity, i.e., the extent to which Internet use affects social and individual quality of life, career, and time control, and excitatory/compensatory usage of the Internet. The score range is 20–100. Considering the Italian cut-off scores (Poli and Agrimi, 2012), Internet usage is defined as non-problematic (scores 20–50), mild to moderate problematic (scores 50–80), and severe problematic (scores 80–100). The reliability of the Italian version is good (α = 0.83), for this study it was α = 0.84.

The Depression Anxiety-Stress Scales (DASS-21; Lovibond and Lovibond, 1995; Italian version by Bottesi et al., 2015) is a 21-item self-report that assesses general distress through three separate subscales (i.e., anxiety, depression, and stress). Scores are considered clinically significant when equal to or over 5 for the depression subscale, equal to or over 4 for the anxiety subscale, and equal to or over 8 for the stress scale (Henry and Crawford, 2005). The reliability of the Italian version is α = 0.85. For this study, the reliability was α = 0.92.

The Barratt Impulsiveness Scale (BIS-11; Patton et al., 1995; Italian version by Fossati et al., 2001) was administered to assess impulsivity. It is a 30-item self-report, with scores ranging from 30 to 120. The higher the score, the higher the impulsiveness level. The reliability of the Italian version is α = 0.89, in this study it was α = 0.75.

The Obsessive-Compulsive Inventory-Revised (OCI-R; Foa et al., 2002; Italian version by Sica et al., 2009) was used to measure obsessive-compulsive symptoms. It is an 18-item self-report that provides separate scores on six subscales, i.e., washing, checking, ordering, obsessing, hoarding, and mental neutralizing, and a total score of Obsessive-Compulsive-related symptoms. Based on the cut-offs of the Italian version, scores in the range of 4–5 on the checking and ordering subscales indicate symptoms bordering on psychopathology that require clinical attention, while scores of 6 and above indicate the presence of clinically significant symptoms. Scores in the range of 3–4 on the washing subscale indicate symptoms bordering on psychopathology that require clinical attention, while scores of five and above indicate the presence of a clinically significant condition. As for the hoarding and the mental neutralizing subscales, scores greater than or equal to 6 and 3, respectively, indicate the presence of a clinically significant condition (Marchetti et al., 2010). The reliability of the Italian version is α = 0.85, in this study it was α = 0.74.

The Alcohol Use Disorders Identification Test (AUDIT; Saunders et al., 1993; Italian version by Addolorato et al., 1999) was used to assess the frequency and quantity of alcohol consumption. Scores range from 0 to 40, with higher scores indicating more problematic alcohol use. Based on Italian cut-offs, a score of 8–12 represents a medium level of alcohol-related problems, whereas scores of 13 and above represent a high level of alcohol-related problems. The Italian version showed good reliability. For this study, the reliability was α = 0.86.

The Cannabis Abuse Screening Test (CAST; Legleye et al., 2011; Italian version by Bastiani et al., 2013) was administered to assess cannabis use with reference to the past 12 months. It is a seven-item self-report, with scores ranging from 0 to 24. Based on Italian cut-offs, scores of seven and above indicate problematic cannabis use. The reliability of the Italian version is α = 0.78, for this study it was α = 0.86.

### Procedure

Upon arrival at the laboratory, participants signed an informed consent form and were asked to complete an *ad-hoc* questionnaire about their demographic characteristics, health status, and medical history, and the self-reports measuring IUD severity, impulsivity, anxiety/depression/stress, obsessive-compulsive symptoms, and use of alcohol and cannabis. The entire procedure took about 30 min.

### Statistical Analysis

To study the relative statistical power of the associations of affective domain-, impulsive-compulsive domain-, and substance abuse-related variables with IUD severity, a multiple regression analysis was employed. The maximum likelihood method was employed to analyze the contribution of statistical predictors in explaining IUD severity, and effect sizes are reported in terms of partial Cohen's f^2^. Multicollinearity was monitored by examining the variance inflation factor (VIF, Craney and Surles, 2002). In this study, the VIF indicated that multicollinearity was not a concern (Impulsivity, *VIF* = 1.27; Anxiety, *VIF* = 2.33; Depression, *VIF* = 2.32; Stress, *VIF* = 2.37; Washing, *VIF* = 1.36; Checking, *VIF* = 2.12; Ordering, *VIF* = 1.85; Obsessing, *VIF* = 2.02; Hoarding, *VIF* = 1.86; Mental neutralizing, *VIF* = 1.36; Alcohol abuse, *VIF* = 1.30; Cannabis abuse, *VIF* = 1.20). Moreover, before running the multiple regression analysis, data were examined for skewness, kurtosis, outliers, and normalcy. The normal Probability-Probability plot of the standardized residuals showed points that were not completely on the line, but close.

Given that the present preliminary study has been run on a convenience sample of 104 Italian young adults, the relative statistical power of the associations of affective domain-, impulsive-compulsive domain-, and substance abuse-related variables with IUD severity was also tested by a Bayesian approach, and the results are reported as Supplementary Material of the present manuscript (see Supplementary Figure 1).

Moreover, a receiver operating characteristic (ROC) analysis was conducted, and the area under the curve (AUC) was calculated as a measure of the accuracy of the diagnostic power of each study variable and each model. The larger the area, the more accurate the diagnostic power, with low AUC in the 0.50–0.70 range, moderate AUC in the 0.70–0.90 range, while an AUC over 0.90 indicates high accuracy (Pintea and Moldovan, 2009).

Intercorrelations among study variables within individuals with mild-moderate IUD and controls were visualized by a network plot (Csárdi and Nepusz, 2006). To test for differences between correlations higher than *r* = 0.30 in individuals with mild-moderate IUD and controls, the correlations were transformed into z-scores using Fisher's r-to-z transformation, and effect sizes were reported in terms of Cohen's q (Cohen, 1988).

All analyses were performed using R software (R Development Core Team, 2016).

## Results

Descriptive statistics and Pearson's correlations are reported in Table 1.

**Table 1:** Descriptive statistics and Pearson's correlations in individuals with mild to moderate IUD and controls.

	**Mean ± sd**	**1**.	**2**.	**3**.	**4**.	**5**.	**6**.	**7**.	**8**.	**9**.	**10**.	**11**.	**12**.
**Individuals with mild-moderate IUD (** ***n*** **= 34)**													
1.Impulsivity	61.24 ± 8.63	1											
2.Anxiety	5.71 ± 3.93	0.32	1										
3.Depression	8.09 ± 5.62	0.19	**0.65**	1									
4.Stress	10.21 ± 4.75	0.15	**0.59**	**0.70**	1								
5.Washing	1.47 ± 1.81	0.00	0.17	0.07	0.10	1							
6.Checking	3.44 ± 3.74	0.06	**0.53**	**0.44**	**0.39**	**0.50**	1						
7.Ordering	4.50 ± 3.30	-0.15	0.14	0.19	0.26	**0.35**	**0.59**	1					
8.Obsessing	5.12 ± 3.33	0.20	**0.67**	**0.62**	**0.62**	**0.41**	0.33	0.22	1				
9.Hoarding	4.15 ± 2.82	0.23	0.32	0.25	**0.50**	0.25	**0.35**	0.30	**0.37**	1			
10.Mental neutralizing	1.71 ± 2.56	–0.14	0.18	0.26	0.27	**0.50**	0.26	0.28	**0.38**	–0.01	1		
11.Alcohol abuse	7.15 ± 4.55	0.32	–0.02	–0.06	–0.04	–0.19	–0.20	–0.07	–0.06	0.01	–0.01	1	
12.Cannabis abuse	1.24 ± 2.73	0.19	0.16	0.20	0.16	0.21	0.16	–0.25	0.25	–0.04	–0.05	0.18	1
**Control group (** ***n*** **= 70)**													
1.Impulsivity	57.91 ± 8.52	1											
2.Anxiety	2.91 ± 2.82	0.14	1										
3.Depression	4.54 ± 3.31	0.14	**0.48**	1									
4.Stress	7.69 ± 4.20	0.03	**0.64**	**0.57**	1								
5.Washing	0.91 ± 1.55	0.03	**0.29**	0.13	0.13	1							
6.Checking	1.76 ± 1.63	–0.21	0	–0.05	0.02	0.14	1						
7.Ordering	2.91 ± 2.47	–0.09	0.22	**0.26**	0.16	**0.26**	**0.48**	1					
8.Obsessing	2.51 ± 2.67	0.20	**0.30**	**0.29**	0.20	**0.32**	0.11	**0.23**	1				
9.Hoarding	1.84 ± 1.71	0.12	**0.27**	0.19	**0.27**	**0.30**	**0.40**	**0.26**	**0.30**	1			
10.Mental neutralizing	0.51 ± 1.48	0.04	0.13	0.07	0.12	0.01	0.12	0.24	**0.32**	0.10	1		
11.Alcohol abuse	5.61 ± 4.79	**0.27**	0.13	**0.27**	–0.01	0.19	0.06	0.10	0.22	**0.33**	0.14	1	
12.Cannabis abuse	0.63 ± 1.63	0.17	0.17	0.07	–0.02	0	–0.09	–0.07	–0.02	0.1	0.03	**0.33**	1

*Bold indicates statistically significant correlations (*p* < 0.05)*.

The multiple regression model including IUD severity as dependent variable and *affective domain-, impulsive-compulsive domain-* and *substance abuse-*related variables showed that only two *impulsive-compulsive domain* variables, i.e., hoarding and obsessing symptoms, and one *affective domain* variable, i.e., depression symptoms, were statistically significant predictors of IUD severity (Hoarding: β = 0.37, *p* < 0.001, *partial f*^2^ = 0.13; Obsessing: β = 0.15, *p* = 0.01, partial *f*^2^ = 0.02; Depression symptoms: β = 0.26, *p* < 0.02, *partial f*^2^ = 0.05). However, the effects of depression and obsessing symptoms were much smaller than those of hoarding symptoms. The regression model explained 40% of the variance (*R*^2^ = 0.40).

**Figure 2:**
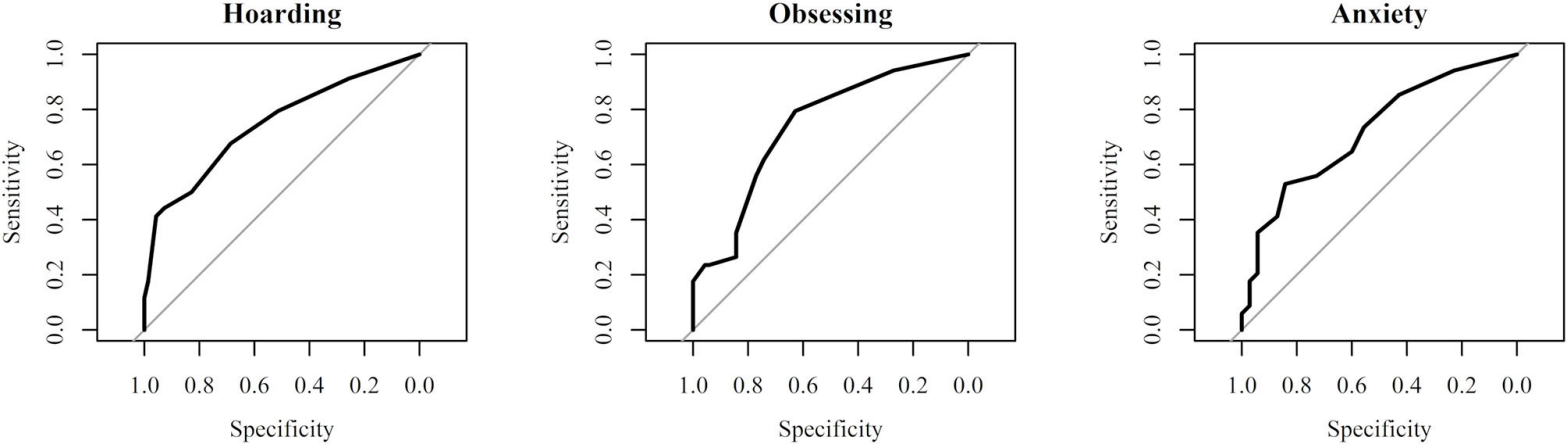
Receiver Operating Characteristic (ROC) plots for hoarding, obsessing, and depression symptoms. Larger areas under the curve indicate better global performance of the variable in discriminating individuals with mild to moderate Internet Use Disorder (IUD) from controls.

When the accuracy of the diagnostic power of each study variable was tested, only hoarding (*AUC* = 0.75), obsessing (*AUC* = 0.74), and anxiety (*AUC* = 0.72) symptoms presented an AUC significantly different from 0.5 (*p* = 0.009, *p* = 0.011, and *p* = 0.03, respectively) and AUC > 0.70, indicating that these *impulsive-compulsive* and *affective domain* variables can distinguish between individuals with mild-moderate IUD and controls (see Figure 2).

**Figure 3:**
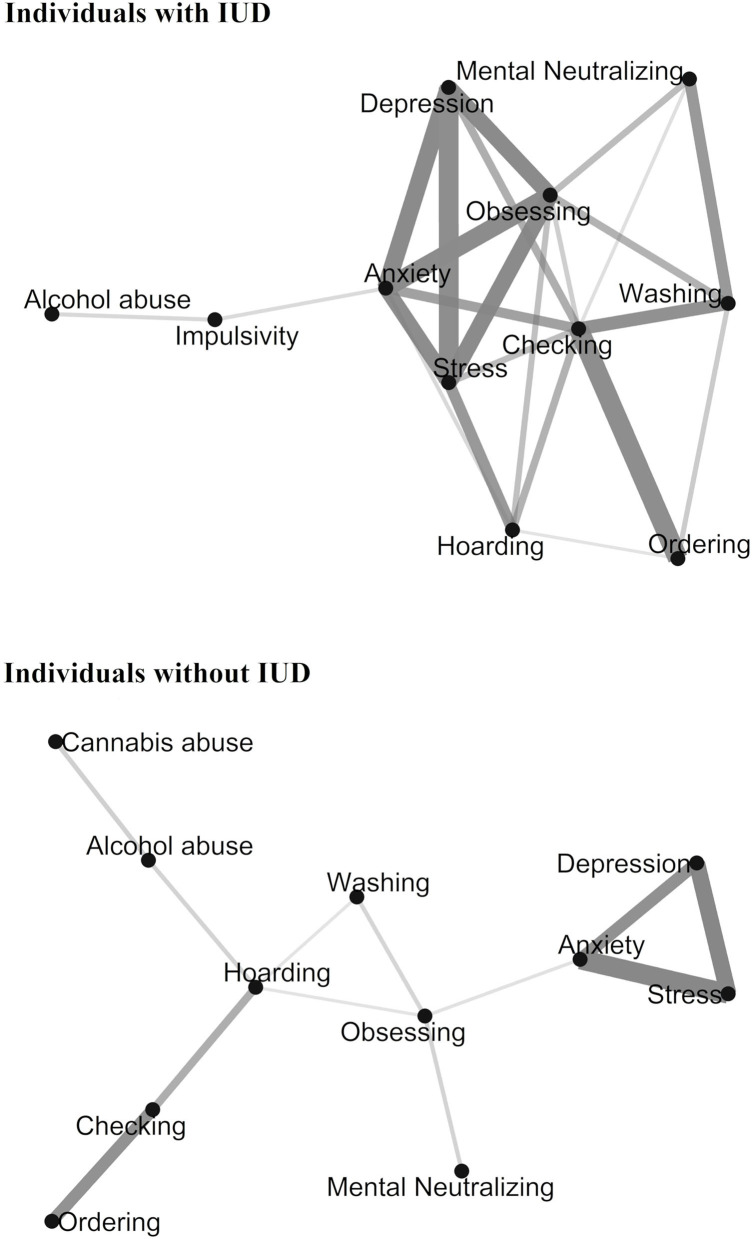
Correlation network plot for individuals with mild to moderate Internet Use Disorder (IUD) and controls. Only Pearson's rs ≥ |0.30| were included. Variables that are more strongly correlated appear closer together and are connected by stronger paths. The proximity of the points is determined using multidimensional clustering.

In the correlation networks among individuals with mild-moderate IUD and controls, correlations were considered as meaningful when *p* < 0.05 and r ≥ |0.30| (Taylor, 1990, see Figure 3). The following correlations were stronger in individuals with mild-moderate IUD than controls: correlations between *affective* and *impulsive-compulsive domain* variables, stress and checking symptoms (z = 1.80, *p* = 0.04, Cohen's *q* = 0.39), stress and obsessing symptoms (z = 2.40, *p* = 0.01, Cohen's *q* = 0.52), anxiety and checking symptoms (z = 2.72, *p* = 0.01, Cohen's *q* = 0.59), anxiety and obsessing symptoms (z = 2.31, *p* = 0.01, Cohen's *q* = 0.50), depression and checking symptoms (z = 1.94, *p* = 0.03, Cohen's *q* = 0.42), depression and obsessing symptoms (z = 1.96, *p* = 0.03, Cohen's *q* = 0.43); and correlations between *impulsive-compulsive domain* variables, washing and checking symptoms (z = 1.88, *p* = 0.03, Cohen's *q* = 0.41), washing and mental neutralizing symptoms (z = 2.48, *p* = 0.01, Cohen's *q* = 0.54).

## Discussion

This study examined potential links between IUD and alterations related to the *affective domain*, the *impulsive-compulsive domain*, and *substance abuse*. As predicted, we found that two *impulsive-compulsive domain* variables, i.e., hoarding and obsessing symptoms, and one *affective domain* variable, i.e., depression symptoms, were positively linked to IUD severity, with hoarding having a higher power to statistically predict IUD severity and greater accuracy to discern individuals with IUD-related problems from controls. While several studies have focused on the relationship between OCD and IUD, this is the first study to our knowledge that investigated specific OCD dimensions in individuals with mild to moderate IUD. Hoarding has been previously reported to have a positive relationship with online/offline compulsive buying (Claes et al., 2016). Of note, despite Hoarding disorder is included among Obsessive-compulsive-related disorders (American Psychiatric Association, 2013), its appetitive aspects, e.g., the pleasure related to the inanimate objects to be hoarded, is thought to be more akin to behavioral addictions (Yap and Grisham, 2019). Moreover, similarly to behavioral addictions, hoarding seems to arise from the anticipation of pleasure and impaired self-regulation (Taylor et al., 2019). Quite recently, “digital hoarding” has been described as a subtype of hoarding disorder, characterized by the accumulation of digital information to the point of loss of perspective, eventually resulting in stress symptoms, with adverse consequences on the individual's functioning in daily life (van Bennekom et al., 2015). If hoarding or, more specifically, “digital hoarding,” is one of the core elements that characterize IUD, and/or if it is a consequence of altered mechanisms that also determine IUD, should be determined given its potential relevance for the diagnosis of IUD. Our finding of a positive link between obsessing and IUD severity fits with previous studies that described obsessing as one of the main factors underlying IUD (Demetrovics et al., 2008). These findings are also similar to what is reported for IGD (Young, 2010), Substance Use Disorders (SUDs; Redish and Johnson, 2007), and sexual and food addiction (e.g., Pelchat, 2002), suggesting that obsessing might be a shared feature between IUD and other addictive behaviors. However, the Bayesian regression did not confirm a relationship between obsessing and IUD severity. Future larger confirmatory studies are needed to clarify this relationship.

Interestingly, among *affective domain-* and *substance abuse*-related variables, the compulsive component of the *impulsive-compulsive domain* seems to be strongly related to behavioral addictions (including IUD and other addictive behaviors), with neurobiological overlaps between SUDs, OCD, and behavioral addictions (Figee et al., 2016). However, evidence of shared dysfunctions in brain activity does not clarify whether these abnormalities are risk factors for IUD, or whether their onset is a consequence of IUD. Since the present study was cross-sectional, it does not allow discussing the results in terms of cause-effect relationships. However, it is noteworthy that a longitudinal study on the precursors and sequelae of IUD in Chinese people found that across several psychopathological symptoms, only OCD symptom levels were higher in Internet addicts than the norm values for Chinese people before these individuals developed Internet addiction. Therefore the Authors argued that OCD could be considered as a predictor for Internet addiction (Dong et al., 2011). Further longitudinal studies are needed to better characterize the timeline of symptom onset in IUD.

With regard to the relationship between IUD and alterations related to the *affective domain*, only depression was significantly positively linked to IUD severity, with a smaller effect size than hoarding. Moreover, anxiety showed a moderate accuracy to discern between individuals with mild to moderate IUD and controls. The associations between altered *affective domain* and IUD have been previously documented (e.g., Li et al., 2019), however, the association of IUD with the interaction between *affective*- and *impulsive-compulsive domain* has been less understood. Of note, while young people with social anxiety have been suggested to use the Internet as a means for interacting with others (i.e., positive reinforcer), young people with obsessive-compulsive symptoms would use the Internet excessively to modulate compulsive anxiety (negative reinforcer; Bernal-Ruiz et al., 2017). Future studies should further explore possible moderating roles of depression and anxiety in IUD as potentially useful variables in the context of clinical assessment and diagnosis formulation.

Overall, these findings suggest that IUD may be characterized by a pattern of symptoms resulting from a disturbance of networks and mechanisms underlying anxiety/mood disorders and OCD. Among such networks, the reward network might play a fundamental role. Functional alterations in the reward network are associated with addiction, depression, and OCD (Park et al., 2019). Similarly to behavioral addictions, it has been shown that both IUD and IGD are related to abnormalities in reward processing, inhibition, and impulse control (e.g., Brand et al., 2016), with increased reward sensitivity and sensitivity to punishment in individuals with IUD (Vargas et al., 2019). These speculations seem to be supported by the findings we obtained when exploring the possible patterns of relationships among *impulsive-compulsive domain, affective domain*, and *substance abuse* variables in the two groups. We found individuals with mild-moderate IUD to be characterized by a pattern of stronger and positive associations of *affective domain* variables with some OCD dimensions than controls. This finding supports the idea of a dysfunctional mechanism shared by altered *affective domain*, OCD, and IUD. Both OCD symptoms and mood disorders would share underlying psychopathological mechanisms, including aberrant activity in reward network/prefrontal-striatal/limbic circuits. As a consequence, maladaptive emotional and behavioral patterns would emerge, including IGD (Han et al., 2018), gambling (Potenza, 2013), and SUDs (Volkow et al., 2010).

Contrary to our expectations, we did not find the impulsivity component of the *impulsive-compulsive domain* to be positively linked to IUD severity. Similar results have been previously reported in some studies that assessed impulsivity in IUD (e.g., Lee et al., 2019). However, other studies have shown that individuals with IUD are characterized by high impulsivity/defective inhibitory control (e.g., Moretta et al., 2019; Moretta and Buodo, 2021). About inconsistent results on the role of impulsivity in IUD, it has been suggested that difficulties in inhibitory control may only emerge in IUD when prepotent responses must be inhibited in an emotional context. Emotional situations would impact behavioral control, with important consequences on effective impulse inhibition in the context of IUD (Moretta et al., 2019; Moretta and Buodo, 2021). We did not find any significant relationship between *substance abuse* variables and IUD severity. Despite the similarities between IUD and substance use disorders (SUDs) in the underlying psychobiological mechanisms, some neurobiological differences between addictions to substances as well as between IGD and SUDs have been extensively described (Park et al., 2017). It may be speculated that IUD shares core psychological and neurobiological aspects of psychostimulants use disorders rather than of Alcohol Use Disorder and Cannabis Use Disorder. Future studies should investigate similarities and differences between IUD and different SUDs.

Overall, our findings suggest that IUD is characterized by a network of interassociated psychopathological symptoms that may reflect a disturbance of mechanisms underlying OCD and affective disorders.

The present study has some important limitations. The first is the small sample size. A small sample size does not allow precise estimates and larger confirmatory studies are needed. Moreover, the sample of the present study was a convenience sample of young adults. Future studies with random sampling methods on other age ranges and cultural contexts are needed. Secondly, it was implemented using a cross-sectional design. This makes it difficult to infer cause-effect relationships between variables. Moreover, results from our exploratory analysis should be considered with caution since they are based on correlations. Third, participants were classified as having a mild to moderate IUD based on Internet Addiction Test scores, such that our sample may not be adequately representative of individuals with IUD. Fourth, we used self-report measures only. Future studies should address our research questions by including behavioral responses to disorder-related stimuli in conjunction with self-reported measures. Lastly, given that the present study is the first to describe the relative contribution of *affective domain, impulsive-compulsive domain*, and *substance abuse* variables to describe IUD severity, there was no related effect size to choose from for a formal power analysis. We believe that this study can be a starting point for considering alterations of *affective domain* and *impulsive-compulsive domain* in IUD for diagnostic and treatment purposes. Future studies are needed to further explore the relationships among these variables in IUD.

Despite these limitations, this study provides new insight into the characterization of IUD, which is the first step for a standardized diagnosis. A standardized and reliable diagnosis is a prerequisite for implementing effective treatments and prevention programs. Our findings may provide preliminary insight for the development of innovative prevention approaches that consider all the psychological aspects described in this study (i.e., *affective and impulsive-compulsive domains*, and *substance addictions*) and their reciprocal relationships. Reaching a consensus regarding the definition, clinical status, and assessment of IUD would upgrade prevention efforts targeting youth significantly, as it would allow the identification of factors that are critical in prevention and intervention programs.

## Data Availability Statement

The raw data supporting the conclusions of this article will be made available by the authors, without undue reservation.

## Ethics Statement

The studies involving human participants were reviewed and approved by Comitato Etico Della Ricerca Psicologica Area 17, Dipartimenti/Sezione di Psicologia, Università degli Studi di Padova, Padova (Italy), comitato.etico.area17@unipd.it. The patients/participants provided their written informed consent to participate in this study.

## Author Contributions

TM: conceptualization data curation, formal analysis, investigation, methodology, project administration, resources, software, visualization, and writing original draft. GB: conceptualization, methodology, resources, supervision, validation, visualization, and review and editing. All authors contributed to the article and approved the submitted version.

## Conflict of Interest

The authors declare that the research was conducted in the absence of any commercial or financial relationships that could be construed as a potential conflict of interest.

## Publisher's Note

All claims expressed in this article are solely those of the authors and do not necessarily represent those of their affiliated organizations, or those of the publisher, the editors and the reviewers. Any product that may be evaluated in this article, or claim that may be made by its manufacturer, is not guaranteed or endorsed by the publisher.
