# Supplementary material for: The Relationship Between Affective and Obsessive-Compulsive Symptoms in Internet Use Disorder
Source: Front Psychol. 2021 Aug 12;12:700518. doi: 10.3389/fpsyg.2021.700518 (PMC8387798; doi:10.3389/fpsyg.2021.700518)

Supplementary material S1

A linear model estimated with Bayesian methods testing the same regression model as described in the main text of the manuscript was performed on the IUD severity using the *rstanarm* (version 2.21.1) R library. The Bayesian regression model was estimated by Monte Carlo Markov Chain (MCMC) procedures with weakly informed priors. The null hypothesis was rejected when the 95% Bayesian credibility intervals (BCIs) did not include the null value.

Similar to what was found by using the frequentist approach, the 95% BCIs showed that higher levels of IUD were associated with higher hoarding (Median = 2.03, 95% BCI [0.848, 3.242]) and higher depression symptoms (Median = 0.74, 95% BCI [0.036, 1.450]). However, in contrast with findings obtained by using the frequentist approach, we did not found evidence supporting an association between IUD severity and obsessing (Median = 0.60, 95% BCI [-0.356, 1.540]). The Median estimates, computed by the MCMC simulation, are reported in Figure S1.

Figure S1. The Median estimates (red line) computed by the MCMC simulation.

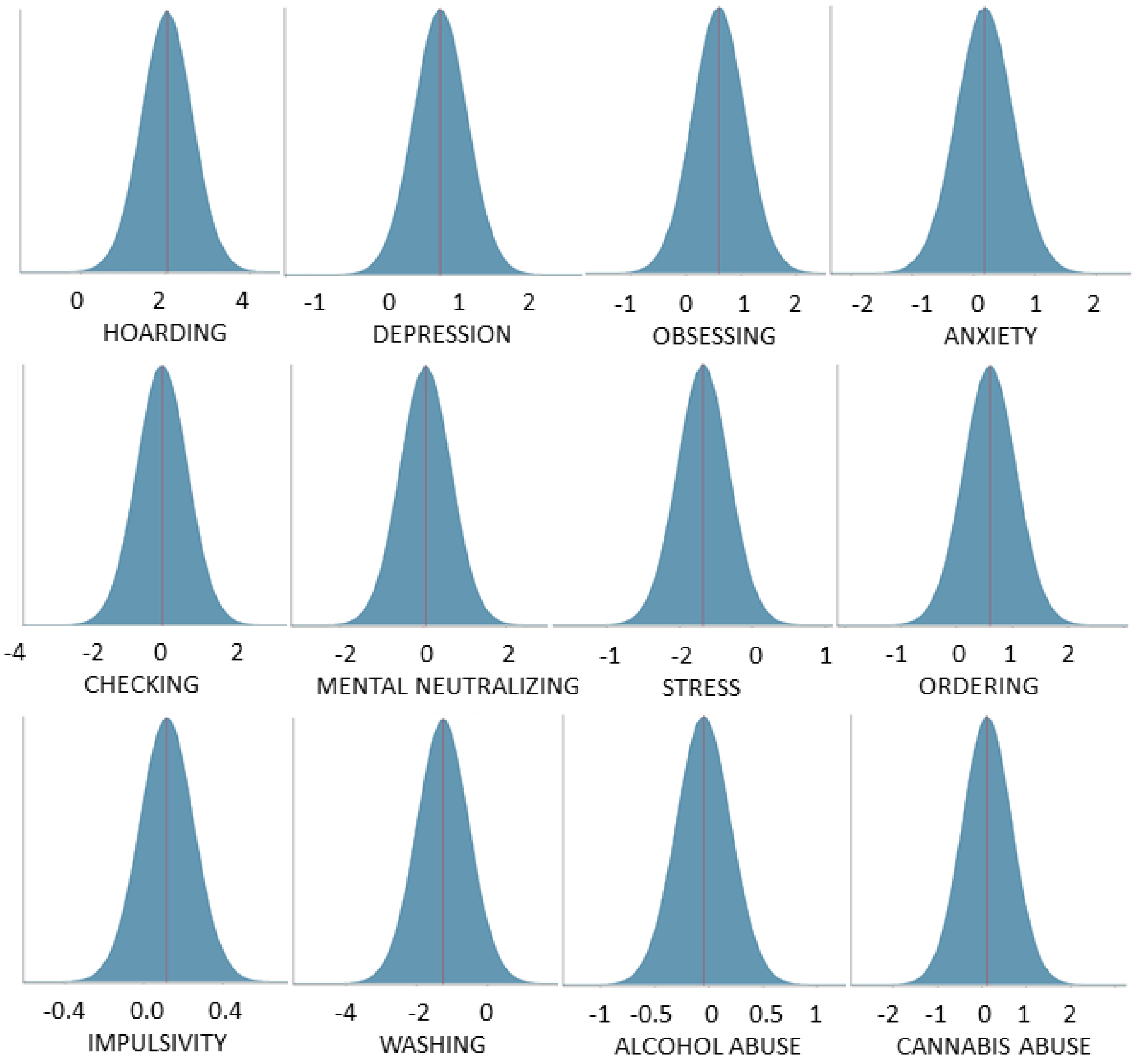

Supplement: Supplementary file 1 [file Data_Sheet_1.pdf]
